# A stochastic frontier analysis of technical efficiency of fish cage culture in Peninsular Malaysia

**DOI:** 10.1186/s40064-016-2775-3

**Published:** 2016-07-19

**Authors:** Gazi Md. Nurul Islam, Shzee Yew Tai, Mohd Noh Kusairi

**Affiliations:** Tun Abdul Razak School of Government (TARSOG), Universiti Tun Abdul Razak, Jalan Tangsi, 50480 Kuala Lumpur, Malaysia; Faculty of Economics and Management, Universiti Putra Malaysia, 43400 UPM, Serdang, Selangor Malaysia; Institute of Agricultural and Food Policy Studies, Universiti Putra Malaysia, Putra Infoport, Serdang, 43400 Selangor Malaysia

**Keywords:** Brackish water, Cage culture, Peninsular Malaysia, Stochastic production frontier, Technical inefficiency, Technical efficiency

## Abstract

Cage culture plays an important role in achieving higher output and generating more export earnings in Malaysia. However, the cost of fingerlings, feed and labour have increased substantially for cage culture in the coastal areas in Peninsular Malaysia. This paper uses farm level data gathered from Manjung, Perak and Kota Tinggi, Johor to investigate the technical efficiency of brackish water fish cage culture using the stochastic frontier approach. The technical efficiency was estimated and specifically the factors affecting technical inefficiencies of fish cage culture system in Malaysia was investigated. On average, 37 percent of the sampled fish cage farms are technically efficient. The results suggest very high degrees of technical inefficiency exist among the cage culturists. This implies that great potential exists to increase fish production through improved efficiency in cage culture management in Peninsular Malaysia. The results indicate that farmers obtained grouper fingerlings from other neighboring countries due to scarcity of fingerlings from wild sources. The cost of feeding for grouper (*Epinephelus fuscoguttatus*) requires relatively higher costs compared to seabass (*Lates calcarifer*) production in cage farms in the study areas. Initiatives to undertake extension programmes at the farm level are needed to help cage culturists in utilizing their resources more efficiently in order to substantially enhance their fish production.

## Background

The marine aquaculture in Malaysia has expanded significantly over the last two decades, contributing about 70 % of the total aquaculture production. Brackish water fish cage culture has received much attention over the years due to its high export demand and generated sizeable foreign exchange earnings for the country. The cage culture areas have increased from 27,000 to 1,741,000 m^2^ between 1982 and 2009. Production has increased from 413 to 22,521 mt during the same period. There were about 3258 farmers involved in cage aquaculture practices in 2009 (Department of Fisheries Malaysia 2011). The Malaysian government took a number of initiatives to promote brackish water cage culture (Ministry of Agriculture 2003). The government has established the Aquaculture Industrial Zones (AIZ) in 2007. Cage culture has been identified as one of the Entry Point Project (EPP) under National Key Economic Area (NKEA) programme which is expected to contribute USD 432 million to the Malaysian Gross National Income by 2020. The floating cages are used as the main production system for marine fin fish. Various species of marine brackish water finfish are produced using the cages, including barramundi or Asian Seabass (*Lates calcarifer*), snappers (*Lutjanidae*), grouper (*Epinephelus fuscoguttatus*), cobia (*Rachycentron canadum*), and red tilapia (*Oreochromis* spp). Among them, barramundi and grouper are the leading species cultured in the coastal areas. Fish cage culture system has been mostly traditional and semi-intensive in Peninsular Malaysia and mostly carried out along the coastal mangroves and relatively shallow lagoons and protected bays. Major fish cage farming has taken place in protected coastal areas in the states of Perak, Johor, Penang and Selangor. The cage culture mainly relies on fish seed or juveniles especially for the grouper collected from the wild. A number of hatcheries have been established in Malaysia to produce seed for various finfish. However, the cost of fry, feed and labour have increased significantly over the recent years. Seabass is a fast growing species which can grow at an average of 1 kg m^−1^ and Tiger grouper can reach marketable size of approximately 0.5 kg m^−1^ within 9–12 months (Rimmer et al. 2005). This size is demanded by consumers in the local market and in neighboring countries. The high value species such as grouper is exported to Hong Kong and China.

Marine commercial cage culture was pioneered in Norway in the seventies with the rise and development of salmon farming (Beveridge 2004). However, cage farming in brackish and inshore waters in Asia is relatively new. Marine and brackishwater cage farming in Asia is diverse, both in terms of variety of species and culture intensities. Phillips and De Silva (2006) reported that the small scale cage culture is highly successful in many parts of Asia but one of the key issues for its continued growth and further development is the management of cage farms (Hambrey 2006). The coastal finfish farming has inadequate supply of fingerlings due to lack of artificial breeding technology. Although China is the largest fish exporter country in Asia but they have problems on breeding technology and culture practices on cage finfish farming (Piumsombun et al. 2005). Cage culture production can be increased through the adoption of improved technology, or through increasing production efficiency by adopting better management and culture systems. Studies have shown that improvement in production efficiency is more cost-effective than introduction of new technologies if the producers are not efficient (Belbase and Grabowski 1985; Dey et al. 2000).

In the stochastic frontier production approach, the technical efficiency is either defined as a minimum set of inputs required to produce a given level of output or alternatively as the maximum output attainable using a given set of inputs (Farrell 1957). Very few studies applied the stochastic frontier analysis in assessing the technical efficiency of the aquaculture sector of Malaysia and of other developing countries. Among the few studies, Sharma (1999), Sharma and Leung (1998), Dey et al. (2000) and Bhattacharya (2009) used the stochastic frontier production function to measure the technical efficiencies of various aquaculture products such as carps, tilapia and shrimp. Similar studies were conducted for aquaculture farms in Taiwan, Philippines, Vietnam and India (Chiang et al. 2004; Irz and McKenzie 2003; Nguyen and Vu 2007; Jayaraman 1998). The main findings of these studies showed that there was a high degree of technical inefficiency among the aquaculture farmers. Iinuma et al. (1999) investigated the technical efficiency of pond culture of carp in Peninsular Malaysia. A similar study was conducted by Sharma and Leung (2000) and they found that productivity of pond culture of carps in Peninsular Malaysia was low and potentials exist for increasing carp production through improved technical efficiency. There is a lack of understanding about the problems in the small scale semi intensive cage farms in tropics region (Beveridge 2004). The output and productivity of cage farming may be affected by external factors such as marine pollution, climate change and other environmental factors which are beyond the control of the culturists. Studies found that there is increased risk of disease occurrence within cage reared fish (Merican 2006; Tan et al. 2006) and the potential risk of transfer of diseases to and from natural fish populations (Ferguson et al. 2007). Further research is required to understand the cage farm management systems (Rimmer et al. 2005).

The objective of this study is to assess the technical efficiency of brackish water finfish aquaculture in floating cage production system in Peninsular Malaysia by applying the stochastic frontier approach. Based on the results of the technical efficiency analysis, some recommendations will be made in order to enhance brackish water fish cage culture production in Peninsular Malaysia.

## Methods

### Stochastic Frontier model

Farrell (1957) described technical efficiency as the ability to produce a given level of output with a minimum quantity of inputs used under certain specific production technology. Aigner et al. (1977) and Meeusen and Van den Broeck (1997) have developed the stochastic frontier production function to measure the technical efficiency of production. The Stochastic Frontier Production Function is more appropriate for measuring technical efficiency because it overcomes the inadequate characteristics of the assumed error term in conventional production functions which have limitations on statistical inference of the parameters and the resulting efficiency estimates.

The stochastic frontier production model can be written as:

1 $$Y_{i} = f(X_{ik} ;\beta_{k} ) + \varepsilon_{i} \quad i = \, 1,2,{ \ldots} n$$

where, Y_i_ denotes the output for the *i*th farm (i = 1, 2, …, n); X_ik_ is a (1 × k) vector of factor inputs of the *i*th farm, and *β*_*k*_ is a (k × 1) vector of unknown parameters to be estimated; ε_i_ is the error term that has two elements, namely:

2 $$\varepsilon_{i} = V_{i} {-}U_{i}$$

where, V_i_ is a random variable which is assumed to be normally, independently and identically distributed, i.e. V_i_ ~ niid(0, *σ*_*ν*_^2^), and independent of the U_i_, and can be positive or negative. The term U_i_ is a non negative random variable which accounts for pure technical inefficiency in production and is assumed to be independently distributed (Aigner et al. 1977). The assumption of the independent distribution between U_i_ and V_i_ allows the separation of the stochastic and inefficiency effects in the model.

Battese and Coelli (1995) defined *U*_*i*_’s as:

3 $$U_{i} = Z_{i} \delta + W_{i}$$

where *Z*_*i*_ is a (1 * p) vector of variables affecting farm efficiency; δ is a (p * 1) vector of parameters to be estimated; *W*_*i*_’*s* represent the truncation of the normal distribution with mean 0 and variance *σ*_*u*_^2^, in such a way that the point of truncation is −*Z*_*i*_*δ*, i.e., *W*_*i*_ ≥ −*Z*_*i*_*δ*. This assumptions are consistent with *U*_*i*_ being a non-negative truncation of the *N*(*Z*_*i*_δ, *σ*_*u*_^2^) distribution.

The maximum likelihood estimation technique is used to simultaneously estimate the parameters of the stochastic frontier model in (1) and those for the technical inefficiency model in (3). The parameters in Eq. (1) include *β’s* and the variance parameters *σ*^2^ = *σ*_*u*_^2^ + *σ*_*v*_^2^ and *γ* = *σ*_*u*_^2^/*σ*^2^ (Battese and Corra 1977), where *σ*^2^ is the sum of the error variance, γ has a value between zero and one, measures the total variation of output from the frontier that attributed to the existence of random noise or inefficiency. Inefficiency is not present when γ = 0 which means that all deviations from the frontier are due to random noise. However, if γ = 1 then the deviations are completely caused by inefficiency effects (Battese and Coelli 1995).

The farm level technical efficiency of production for the *i*th farm (TE_*i*_) is defined as:

4 $$TE_{i} = \exp ( - U_{i} ) = \frac{{Y_{i} }}{{f(X_{i} ;\beta )\exp (V_{i} )}}$$

The prediction of the technical efficiency is based on the conditional expectation expressed in (4), given the model specification (Battese and Coelli 1988).

### Empirical Model Estimation

The Cobb-Douglas stochastic production frontier model has been commonly used in many aquaculture studies in developing countries (Iinuma et al. 1999; Nerrie et al. 1990; Hsiao 1994). This model will be used in the specification of (1) as follows:

5 $$LnY_{i} = \beta_{0} + \beta_{1} Ln \, X_{1} + \beta_{2} Ln \, X_{2} + b_{3} LnX_{3} + b_{4} LnX_{4} + b_{5} LnX_{5} + b_{6} LnX_{6} + V_{i} {-}U_{i}$$

where *Ln* is the natural logarithm, Y_=_ fish production (kg); β_1_, β_2_, β_3_, β_4_, β_5_, and β_6_ are the regression coefficients of inputs (input elasticities); X_1_ = fish fry (pieces); X_2_ = feed (kg); X_3_ = labour (days); X_4_ = fuel (liter); X_5_ = utility (USD); X_6_ = other maintenance (USD); and V_i_ + U_i_ are the error terms. The definitions, measurements and summary statistics of all the variables in (5) are presented in Table 1. Maximum likelihood estimation of (5) provides the estimates for the *β’s* and the variance parameters, *σ*^2^ = *σ*_*v*_^2^ + *σ*_*u*_^2^ and *γ* = *σ*_*u*_^2^/*σ*^2^. The empirical specification for the random variable associated with technical inefficiency as in (3) is shown in (6) below:

6 $$U_{it} = \delta_{0} + \delta_{1} Z_{i1} + \delta_{2} Z_{i2} + \delta_{3} Z_{i3} + \delta_{4} Z_{i4} + \delta_{5} Z_{i5} + \varepsilon$$

where U_it_, δ and ε are as defined earlier. The variables of Z_1_ = age (year); Z_2_ = Education (year); Z_3_ = Experience (year); Z_4_ = Production cycle (number); and Z_5_ = Cage area (meter square). The summary statistics of these variables included in the model is presented in Table 1.

**Table 1:** Summary statistics for variables included in the Stochastic frontier production and technical inefficiency models for cage culture

Variable name	Definition	Measurement	Summary statistics			
			Mean	SD	Min	Max
Output and input variables						
Y	Cage fish production per cycle	(kg m^−2^)	9.4	27.5	0.02	232
X_1_	Fish fry	(pieces m^−2^)	48.8	109.8	0.29	619
X_2_	Feed	(kg m^−2^)	58.6	211.9	0.03	1332
X_3_	Labour (own and hired)	(days m^−2^)	1.8	3.2	0.02	21.3
X_4_	Energy (fuel)	(litre m^−2^)	19.2	42.3	0.00	241.9
X_5_	Utility (operational)	(USD m^−2^)	6.9	10.8	0.00	68.8
X_6_	Others (maintenance)	(USD m^−2^)	0.5	0.6	0.00	3.1
Farm specific variables						
Z_1_	Age	(year)	48	11	28	78
Z_2_	Education	(year)	8	4	0	19
Z_3_	Experience	(year)	7	7	1	33
Z_4_	Production cycle	(number)	1.7	0.88	1	5.5
Z_5_	Cage area	(m^−2^)	1127	1847	36	11,250

From the model estimation results, the output for each farm can be compared with the frontier level of output given the level of inputs employed. This deviation indicates the level of inefficiency of the firm. Therefore, the technical efficiency score for the *i* th farm in the sample (TE_i_), can be defined as the ratio of observed output to the corresponding frontier output (Coelli et al. 2005), that is:

7 $$TE_{i} = { \exp }\left( { - U_{i} } \right)$$

where TE_i_ is the technical efficiency of the farm (0 < TE < 1). When *U*_*i*_ = 0 then the *i*th farm lies on the stochastic frontier and is known as technically efficient. If *U*_*i*_ > 0, the farm *i* lies below the frontier, which means that the farm is inefficient. The model has been estimated using Limdep 7.0 software.

### Data and variables

The data for the study were collected from a survey of fish cage culture farmers. The sample of fish cage culture farmers was selected from two major fish cage culture producing States in Peninsular Malaysia namely, Perak and Johor. The respondents were randomly selected from among the fish cage culture farmers within each state: 14 farmers from the *Manjung* district of Perak and 64 from the *Kota Tinggi* district of Johor. The selected respondents were either owner or manager of fish cage farming. Data for cage culture activities were collected through face to face interviews of the sampled respondents using a structured questionnaire during the months of October and November of 2010. The information collected through the questionnaire include the demographic characteristics of cage culturists; the physical characteristics of culture system and type of species stocked in cage farms; inputs used and cost of production; and the quantity and value of fish production.

The study obtained data on inputs used such as seed (fish fry), feed (kg), labor (days), fuel (liter), operational expenditure (USD), maintenance cost (USD). The study measure all the inputs used in a cycle in the form of quantity per total size of cages (m^−2^). Labor is measured in number of labor employed multiply number of farming days per cycle, then divided by the size of cage area (m^−2^). Feed is measured in kilogram of trash fish and pellet divided by size of cage (m^−2^). Seed is measured in number of stocked fry divided by size of cage (m^−2^). Energy cost is measured in USD for cost of fuel for travelling to the cage. Operational expenditure is measured in USD by total expenses for other operational costs. Other maintenance costs is measured in USD spent on maintenance of cages and nets. The study obtained demographic variables which include farmers age (years), education level (year), experience in cage aquaculture (years), number of production cycle in a year (number) and number of cages operated per farmer (number). The definitions, measurements and the summary statistics for variables used in this study are presented in Table 1.

## Results

### Sample characteristics

The average farm size of cages was 1127 m^2^; average size of cage farms was relatively larger in Perak (2159 m^2^) compared to Johor area (902 m^2^). The average fish production was 9.4 kg m^−2^; average production was 7.5 kg m^−2^ in Perak while 9.8 kg m^−2^ in Johor. Production cycle for grouper finfish requires relatively longer time (about a year) to grow compared to the sea bass which require about six months. The average feed costs per cycle was USD 14.1 m^−2^, with cages in Perak having relatively higher feed costs (USD 31.2 m^−2^) compared to those in Johor (USD 10.4 m^−2^). Both seabass and grouper farming require intensive labour, for chopping trash fish prior to feeding and cleaning the nets, dipping of fish in freshwater to prevent disease. Average number of labour days employed was 1.8 man-days in a single production cycle. All cage farmers purchased fish fingerlings from local hatcheries. The average cost of fry was USD 17.5 m^−2^. Sea bass farmers obtain fingerlings from local hatcheries, while grouper farmers obtain fingerlings from both local sources and from Thailand, Indonesia and Philippines.

The maximum likelihood (ML) estimates of the parameters in Eqs. 5 and 6 are presented in Table 2. The results show that all the estimated *β* coefficients have positive signs except that for the costs of other inputs and maintenance costs. Fingerlings and labour are the two inputs significantly different from zero at 1 % level which imply that these inputs have major influence on the production of fish cage system in Peninsular Malaysia. The coefficient for feed is significant at 10 % level which indicates that feed has also influence on fish production in marine cage culture. The coefficient for other input costs and maintenance costs are negative but does not significantly correlate with cage production of fish.

**Table 2:** Parameter estimates of stochastic production frontier and technical inefficiency models

Variable	Parameter	Coefficients	Standard error
Stochastic production frontier			
Constant	β_0_	1.1236*	0.4424
Ln seed (fish fry)	β_1_	0.3984***	0.1157
Ln (Feed)	β_2_	0.1897*	0.0761
Ln (Labour)	Β_3_	0.3239***	0.1194
Ln (Operational costs)	β_4_	−0.0714	0.0736
Ln (Energy)	β_5_	0.0869	0.0799
Ln (Other inputs)	β_6_	−0.0435	0.1009
Technical inefficiency model			
Constant	δ_0_	0.4535	0.1729
Age (no. of years) of respondent	δ_1_	−0.0029	0.0025
Experience (no. of years)	δ_2_	−0.0028	0.0042
Cage area (m^−2^)	δ_3_	−0.0177	0.0169
Education (level)	δ_4_	−0.0024	0.0074
Production cycle (number)	δ_5_	−0.1099**	0.0349
Variance parameter			
Sigma-square *σ*_*s*_^2^ = *σ*_*u*_^2^ + *σ*_*v*_^2^		1.9947**	0.0187
Gamma *γ* = *σ*_*u*_^2^/*σ* ^2^		0.9774***	3.0008
Log likelihood		−118.86	
Mean of exp (−U_i_)		0.37	

*** Estimates are significant at 1 %, ** significant at 5 %, * significant at 10 %

The results for the estimation of the technical inefficiency model’s parameters show that the coefficient for age, experience, education and number of production cycle are negative but not significant except the number of production cycle which is significantly different from zero at 5 % level. The result implies that an increase in age, cage culture experience and an increase in the level of formal education of cage culture owners/managers will reduce technical inefficiencies in the fish cage farming. The increase in the number of production cycle will significantly reduce technical inefficiencies. Majority of cages with sea bass undertake two production cycles a year but only one cycle per year is normally carried out for grouper since it requires 7–12 months per cycle for the growth of the species. The results suggest that increasing harvest frequencies will be more technically efficient as the growth of these species are slow.

**Fig. 1:**
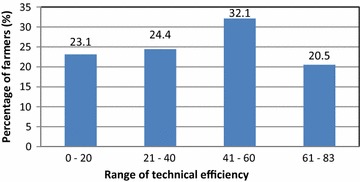
Frequency distribution of technical efficiency for fish cage culture in Peninsular Malaysia. *Note*: Mean technical efficiency for first group is 0.08 (range 0.02–0.16), 0.31 (range 0.21–0.40) for second group, 0.52 (range 0.41–0.60) for third group, and 0.69 (range 0.61–0.83) for the last group

The value of the gamma coefficient is 0.977 and is significant at 1 % level (Table 2). This shows that the output variations among the fish cages are dominated by technical inefficiency rather than random shocks. The predicted technical efficiencies (TE) of all the sampled farms range from 0.174 to 0.861 with the mean technical efficiency of 0.375 (Fig. 1). This indicates that if fish cage farmers use their existing level of inputs in an efficient manner, output on average can be increased by 63 %. This result suggests that the potential for increasing fish production in Peninsular Malaysia through improved technical efficiency is rather substantial. If all sampled farmers are able to achieve the level of output of its most efficient counterpart, then a 62 % [i.e., 1 − (37.5/97.7) × 100] saving on inputs use could be realized, and the most technically inefficient farm could achieve saving on inputs used by 82 % [i.e., 1 − (17.4/97.7) × 100]. The results indicate that the technical inefficiency has significant impacts on the levels and variations of fish production in cage systems in Peninsular Malaysia. The distribution of the predicted efficiency levels of fish cages production system at the study sites is presented in Fig. 1. It can be observed from the figure that more than 32 % of the cage farmers operate their farms within the efficiency of 41–60 % range.

Table 3 shows the results of technical efficiency by farm size. The results of the study show that the technical efficiency is not significantly different between the large and small farms (0.37 and 0.36). The results do not support other studies where the authors have found that technical efficiency was significantly different between large and small brackish water pond culture systems (Kumar et al. 2004; Dey et al. 2000; Irz and McKenzie 2003).

**Table 3:** Technical efficiency for fish cage culture in Peninsular Malaysia by farm size and operation status categories

Variable	Technical efficiency (%)
Farm size	
Small farm (<999 m^2^)	36
Large farm (≥1000 m^2^)	37
Operation status	
Owner-operator	36
Non owner-operator	40

In terms of the ownership and operational status of the farms, the results show that owner operators were relatively less efficient (0.36) compared to non-owner operators (0.40), however the level of efficiency is not significantly different between the operators. The lower technical efficiency for both owner and non-owner operated farms could be due to the fact that these farms were managed by farmers with traditional technical management knowledge and skills. Other study of shrimp farming in India found that leased farms are relatively less efficient than owned farm does not support to this result (Kumar et al. 2004).

## Discussions

The results of the study reveals that the mean technical efficiency for sample cage farms is estimated to be about 37 %. The results suggest that potentials exist to increase fish production through improved technical efficiency in cage culture management in Peninsular Malaysia. The results of the study show that seed and labor were the important inputs towards fish production in marine cage culture. The cost of production for cage farms is substantially higher in Perak compared to Johor area. Farmers obtain wildcaught grouper fry from the nearest locations in Johor and used low cost trash fish as feeding for the finfish. In Perak, wild fry is not available, majority used pelleted feed and seabass fry in their cages imported from other countries. These results have been supported by studies in other small scale semi intensive cage farms in Asia where cage farming systems are facing problems with inadequate seed and feed (Phillips and De Silva 2006).

The results of technical inefficiency model indicate that farmers experience and the number of production cycles were negatively related to technical inefficiency of finfish cage culture in the study areas. This imply that technical inefficiencies of cage farms could be reduced significantly through increasing the number of production cycle. The slow growth of grouper in cage farms has been identified as the main difficulty to increase production in Malaysia and other countries in South East Asia. The growth of grouper depends on the type of seed, feed and feeding practices in cage culture. Farmers mainly use trash fish due to low cost and locally available feed for grouper compared to pelleted feed. It is generally believed that feeding trash fish can improve the texture and appearance of the fish that can fetch high market value. The results reveal that farmers experience is lacking in cage culture activities. Improving skills in cage culture management will reduce the inefficiency of cage farms in Malaysia. Study found that farmers have lack of knowledge and skills in the daily care and feeding activities required in cage culture in Indonesia (Ahmad and Sunyoto 1990).

The evidence suggest that the cage farms are technically inefficient due to poor knowledge in cage aquaculture in Malaysia. Farmers are dependent on wildcaught seed because of the low survival rates of grouper fingerling raised in marine cage farming in Asia (De Silva and Phillips 2007). Several studies found that the trash fish are often insufficient and unreliable in quality and quantity to meet demand, and large scale cage aquaculture is not possible (Ruangpanit 1999; Yashiro et al. 1999; Yongzhong 1999). Farmers mostly rely on wildcaught grouper seed in Malaysia. However, most of the grouper fry is collected from the coastal areas of Johor state. The farmers of Johor obtains grouper seed from the local areas, sea waters are relatively clean compared to the Perak areas where farmers have been involved in various aquaculture systems in the coastal areas over the past several years. There is potential to expand high value fast growing grouper culture in Johor state in Peninsular Malaysia. However, perceptions regarding the poor adaptability, relatively slow growth rates compared with low-value finfish, and poor availability of pellets need to be overcome (De Silva and Phillips 2007).

## Conclusions and recommendation

The mean technical efficiency of finfish farms is estimated to be 37 %. This suggests that great potential exists for increasing cage production in Peninsular Malaysia through improving technical efficiency of brackish water cage culture of finfish. In Malaysia, cage culture in the coastal areas are small scale, traditional and semi intensive. The results of the study show that farmers used wildcaught seeds and trash fish feed for the grouper and hatchery seeds and pelleted feed for seabass in cage farms.

The results show that production of cage culture with grouper requires relatively longer time to grow. The slow growth of grouper with greater reliance on wildcaught fingerlings are the barriers to operate large scale cage finfish production. Increased research effort and funding should be directed to overcome the problem of inability to produce fast growing other grouper seeds through breeding technology and to enhance the quality seed supply from local hatcheries.

The results suggest that there is lack of skills and knowledge of cage farmers in feed management. Feed accounts for the major costs of finfish aquaculture. Further research could help in developing other formulated feed which is acceptable for cage farmers in Malaysia. The formulated feed may increase growth of cultured fish and save time in the culturing period. In order to increase the technical efficiency and enhance productivity in cage farms, information dissemination through extension and trainings should be provided to cage farm owners/managers to enhance their skills and knowledge related to the importance of quality seeds and reduction in feed costs.

The study covered only two of eleven states in Peninsular Malaysia. Similar studies in different geographical locations in Malaysia would provide more detail and comprehensive information on the level of technical efficiency in finfish cage aquaculture in Malaysia.
